# Treadmill exercise promotes E3 ubiquitin ligase to remove amyloid β and P-tau and improve cognitive ability in APP/PS1 transgenic mice

**DOI:** 10.1186/s12974-022-02607-7

**Published:** 2022-10-04

**Authors:** Longfei Xu, Mingzhe Li, Aili Wei, Miaomiao Yang, Chao Li, Ran Liu, Yuejun Zheng, Yuxin Chen, Zixi Wang, Kun Wang, Tianhui Wang

**Affiliations:** 1 Institute of Environmental and Operational Medicine, Academy of Military Medicine Sciences, Academy of Military Sciences, 1 Dali Road, Heping District, Tianjin, 300050 People’s Republic of China; 2 Tianjin Key Laboratory of Exercise Physiology & Sports Medicine, Tianjin University of Sport, Tianjin, 301617 People’s Republic of China

**Keywords:** Physical exercise, E3 ubiquitin ligase, Alzheimer’s disease, Amyloid-β, Tau, Cognitive function

## Abstract

**Background:**

Moderate physical exercise is conducive to the brains of healthy humans and AD patients. Previous reports have suggested that treadmill exercise plays an anti-AD role and improves cognitive ability by promoting amyloid clearance, inhibiting neuronal apoptosis, reducing oxidative stress level, alleviating brain inflammation, and promoting autophagy–lysosome pathway in AD mice. However, few studies have explored the relationships between the ubiquitin–proteasome system and proper exercise in AD. The current study was intended to investigate the mechanism by which the exercise-regulated E3 ubiquitin ligase improves AD.

**Methods:**

Both wild type and APP/PS1 transgenic mice were divided into sedentary (WTC and ADC) and exercise (WTE and ADE) groups (*n* = 12 for each group). WTE and ADE mice were subjected to treadmill exercise of 12 weeks in order to assess the effect of treadmill running on learning and memory ability, Aβ plaque burden, hyperphosphorylated Tau protein and E3 ubiquitin ligase.

**Results:**

The results indicated that exercise restored learning and memory ability, reduced Aβ plaque areas, inhibited the hyperphosphorylation of Tau protein activated PI3K/Akt/Hsp70 signaling pathway, and improved the function of the ubiquitin–proteasome system (increased UCHL-1 and CHIP levels, decreased BACE1 levels) in APP/PS1 transgenic mice.

**Conclusions:**

These findings suggest that exercise may promote the E3 ubiquitin ligase to clear β-amyloid and hyperphosphorylated Tau by activating the PI3K/Akt signaling pathway in the hippocampus of AD mice, which is efficient in ameliorating pathological phenotypes and improving learning and memory ability.

**Supplementary Information:**

The online version contains supplementary material available at 10.1186/s12974-022-02607-7.

## Background

Alzheimer’s disease (AD) is a group of age-related progressive neurodegenerative diseases and is the most common form of dementia. The World Alzheimer’s Disease Report 2019 states that there are currently at least 50 million people with dementia worldwide, and by 2050, the number of people with dementia is projected to be 152 million [1], which has major implications for individuals, families, and health care systems. Therefore, it is increasingly important to conduct more research on the prevention and treatment of AD. The main pathological feature of AD is the presence of a large number of senile plaques with extracellular Aβ as the main component in the cerebral cortex and hippocampus senile plaque (SP) and neurofibrillary tangles (NFTs) with hyperphosphorylated Tau (P-Tau) as the main component, leading to severe impairment of neuronal function and synaptic capacity in specific brain regions, cognitive decline and memory impairment [2]. The degradation of intracellular and/or extracellular abnormal proteins is mainly accomplished by the ubiquitin–proteasome system (UPS) to restore protein homeostasis and maintain all cellular functions. Dysfunctional UPS is associated with the accumulation of Aβ in AD and Tau protein hyperphosphorylation, causing proteostasis imbalance.

UPS-related proteins, such as carboxyl terminus of HSC70 interacting protein (CHIP) and ubiquitin carboxyl-terminal esterase-1 (UCHL-1), play important roles in AD, and their abnormal expressions lead to UPS dysfunction in AD patients [3, 4]. CHIP can act as a co-chaperone of heat shock protein 70 (HSP70) that interacts with HSP70 to form a mediated CHIP/HSP complex leading to ubiquitination of misfolded proteins and assisting in the presentation of target proteins to the proteasome for degradation [5]. In animal models of AD, reduced expressions of HSP70 [6] and deletion of CHIP [7] correspondingly lead to accumulation of hyperphosphorylated tau proteins, suggesting that damage to the folding pathway plays a key role in neurodegeneration and accelerates the progression of AD. In addition, Heat shock factor 1 (HSF1) is a key regulator of induced HSP70 expression, and in AD brains, HSF1 activity and protein expressions are reduced [8], suggesting that the upstream factors of HSP70 are inhibited, that a corresponding cascade response exists to regulate abnormal protein folding. Abnormal function of UCHL-1 and inhibition of ubiquitin hydrolytic activity of UCHL-1 in AD mice disrupt the distribution of synaptic proteins increase the spine size of hippocampal neurons, decrease spine density, and exacerbate cognitive impairment [9, 10]. The expression of UCHL-1 is down-regulated in early AD brains [11], and is involved in the degradation of APP and β-secretase 1 (BACE1). The reduced UCHL-1 content leads to impaired APP and BACE1 degradation and increases the amount of BACE1 [12], which results in Aβ overproduction. Recent research shows that the phosphatidylinositol 3-kinase/protein kinase B (PI3K/Akt) signaling pathway is critical to the physiological and pathological conditions of AD [13]. In the hippocampus of AD transgenic mice, there is an abnormal PI3K/Akt signaling pathway with decreased levels of phosphorylation of PI3K and Akt [14] and suppressed expressions of HSP70 [15, 16], accompanied by increased neuronal loss and apoptosis and increased inflammatory response. In neuronal cells, changes in PI3K/Akt pathway activity modulate HSP70 expressions [17], suggesting that the PI3K/Akt signaling pathway regulates HSP70 translocation, and that HSP70 is a downstream factor of the PI3K/Akt signaling pathway.

In recent years, exercise as a healthy lifestyle has reduced the risk of AD to some extent. Numerous studies have shown that long-term regular exercise can reduce Aβ [18] and hyperphosphorylated Tau protein levels [19], improve the cognitive level of AD patients [20], and effectively delay the onset of AD. Moreover, aerobic exercise has been shown to activate the PI3K/Akt signaling pathway in the brain of AD transgenic mice to upregulate HSP70 protein levels [21], thereby exerting a neuroprotective effect. However, the exact mechanism by which exercise regulates the expression of E3 ligases (CHIP, UCHL-1) to affect UPS function is not clear in the AD state, which was why this study was conducted in APP/PS1 mice to find out whether exercise could improve the pathological phenotype of AD by affecting the PI3K/Akt pathway and thus altering the expression of E3 ubiquitin ligase. This research focused on the effects of exercise on cognitive ability, Aβ plaque areas, Tau protein hyperphosphorylation content, and E3 ubiquitin ligase expression levels.

## Materials and methods

### Animals

Three-month-old male APPswe/PS1dE9 (APP/PS1) double transgenic mice [B6-Tg (PrP- hAPP/h PS1)] and their age- and background-matched wild-type (WT) mice [C57BL/6] were purchased from the BEIJING HFK BIOSCIENCE CO., LTD, (Beijing, China). The successful modeling of APP/PS1 transgenic mice could be confirmed by the test report (No. ZG-3.5-02). Mice were housed in pairs in cages and had access to food and water ad libitum. The facility environment had controlled light (12:12 h light/dark cycle), relative humidity (50 ± 10%) and temperature (22 ~ 25 °C) under conventional laboratory conditions. APP/PS1 transgenic mice were randomly assigned to two groups (*n* = 12 for each group): transgenic control (ADC) and transgenic exercise (ADE). Similarly, wild-type littermate mice were randomly divided into two groups (*n* = 12 for each group): wild-type control (WTC), wild-type exercise (WTE). All experimental procedures were approved by the Laboratory Animal Welfare Ethics Committee of the Institute of Environmental and Operational Medicine. All efforts were made to minimize the number and suffering of animals used in these experiments.

### Treadmill exercise protocols

**Fig. 1:**
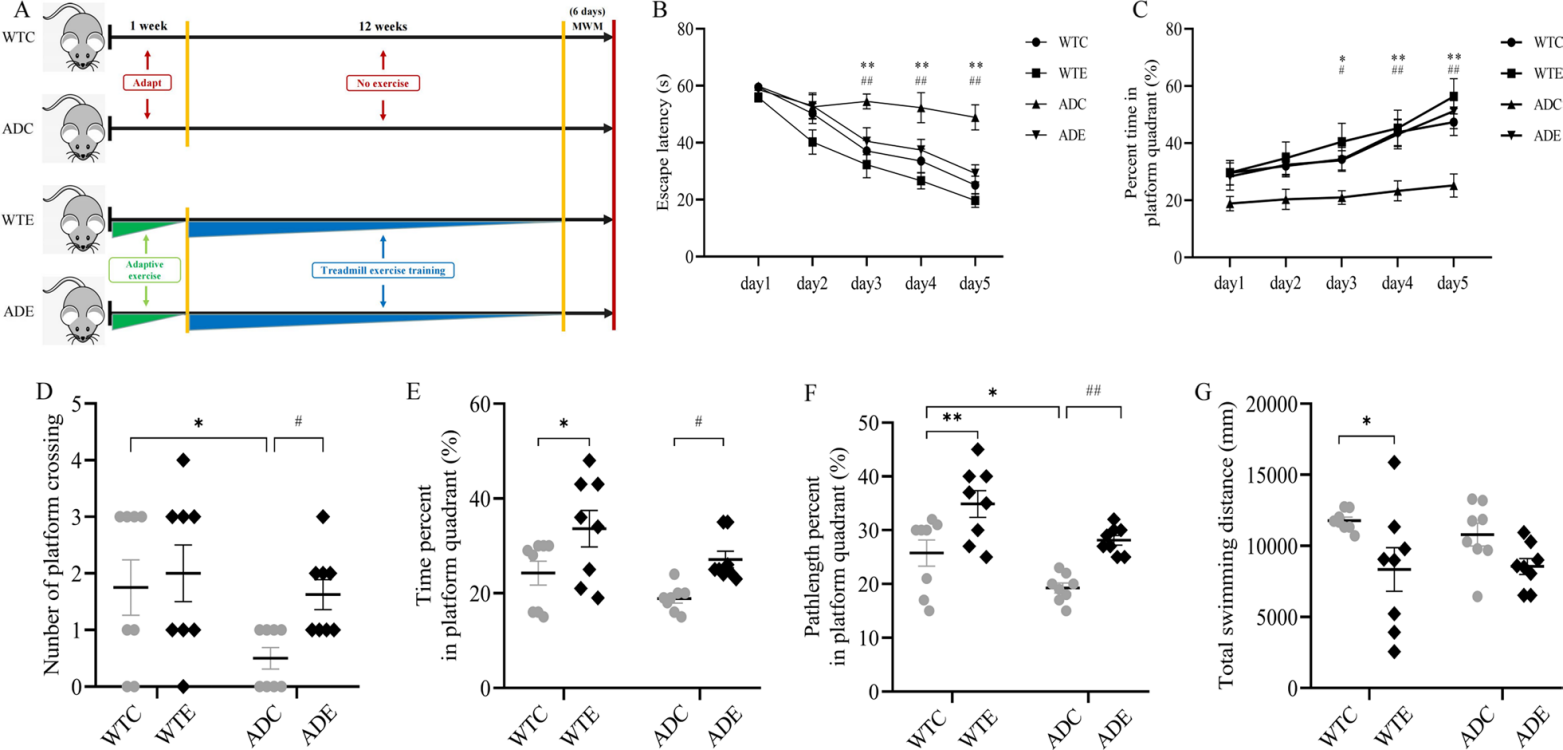
Effect of treadmill exercise on cognitive functions assessed with the Morris water maze (*n* = 8 for each group). **A** Schematic diagram of the exercise protocol in mice. **B**, **C** Mean escape latencies to reach the platform and average percentage of time spent in the platform quadrant for 5 consecutive days during the navigation test. **D, E** Numbers of platform crossings and percentage of time spent in the platform quadrant during the probe test on day 6. **F, G** Percentage of pathlength in the platform quadrant and total swimming distance during the probe test on day 6. Values are presented as mean ± SEM. In the navigation test and probe test, statistically different from WTC, *P < 0.05; **P < 0.01. Statistically different from ADC, ^#^*P* < 0.05; ^##^*P* < 0.01. ADE transgenic exercise, ADC transgenic sedentary, WTE wild-type exercise, WTC wild-type sedentary

The exercise protocol in this experiment referred to Baker’s research [22] and made appropriate modifications. 45%-55% maximal oxygen uptake (VO_2max_) was used as the exercise intensity of this experiment, which was an appropriate intensity of long-term aerobic exercise. Mice in WTE and ADE groups received 6-day adaptive training (familiarity period, 15 min/day) to adapt to the new environment. On the first and second days, the treadmill was engaged to a walking speed of 5 m/min before the speed was increased to 8 m/min on the third and fourth days and 12 m/min on the fifth and sixth days. Mice in the exercise groups received formal training on a treadmill with a frequency of 5 days per week for 12 weeks. In each session, mice ran on the treadmill at zero inclination at a speed of 5 m/min for 5 min and then 8 m/min for 5 min (warming up), after which the speed was increased to 12 m/min for 30 min (main exercise), and finally at 12 m/min for 5 min (recovery). Mice assigned to ADC and WTC groups were placed on the static treadmill for a matched stage to minimize the impact of the experimental environment on the results during the entire training (Fig. 1A).

### Morris water maze test

After the 12-week exercise regimen, the capacity of learning and memory of eight mice from each group was evaluated with a Morris water maze (MWM) test as described previously [23]. Firstly, an appropriate amount of thermostatic water (24 ~ 26 °C) was added to the circular pool, and the height of the water exceeded the platform in the pool by 1–2 cm. The pool was divided into four quadrants and the platform was placed in the first quadrant as the target quadrant. An appropriate amount of skimmed milk powder was sprinkled into the pool to keep the water turbid. Before the initiation of the MWM test, all mice received a 1-day familiarization session to ensure that they could swim and find a round platform. During the place navigation test, the mice were tested for 5 consecutive days (by entering water from fixed positions in quadrant1, 2, 3, and 4) to find the platform (constant position) so as to evaluate the acquisition ability of spatial learning and memory of mice. If the mice failed to find the platform within 60 s, the escape latency was recorded as 60 s, and the mice were placed on the platform to learn for 10–15 s. During the space probe test (day 6), the platform was removed from the maze and the mice were placed in water from four quadrants in sequence to find the location of the platform within 60 s so as to assess their memory retention ability of the platform location. A camera above the pool was used to capture images of the mice swimming during the test. These images were analyzed with a computerized video-tracking system (Xinruan Information Technology Co., Ltd, Shanghai, China) to determine several key indicators: (a) escape latency (time taken to reach the escape platform), (b) percentage of the time spent in the platform quadrant, (c) the number of platform crossings, (d) percentage of pathlength in the platform quadrant, (e) total swimming distances, and (f) path trajectory. In all trials, mice were released at the edge of the pool, facing towards the wall (Additional file 1).

### Tissue preparation

Twenty-four hours after the MWM test, all mice fasted for 12 h. Three mice in each group (no behavioral tests) were anesthetized and transcardially perfused with 1 × PBS (PH 7.4). The whole mouse brains were excised and fixed in 4% paraformaldehyde for immunofluorescent staining. The rest of the mice in each group were anesthetized and decapitated, their brains were dissected, and left and right hippocampi were collected separately and stored at – 80 °C until Western blotting and real-time PCR. Dissection of the whole brain and separation of the hippocampus were performed on ice.

### Immunofluorescence staining and quantification

The brain tissue was dehydrated, embedded and cut to make paraffin sections. Three sections were selected between 1.70 mm and 2.70 mm posterior to bregma, and cut into 4-μm coronal sections for Immunofluorescence. The tissue sections were placed in a repair box filled with citric acid antigen repair buffer (pH 6.0) or EDTA antigen repair buffer (pH 9.0) for antigen repair in a microwave oven before they were cooled to room temperature. Sections were immersed with 5% BSA (bs-0292P, Bioss Biotechnology Co., Ltd. Beijing, China) for 60 min at room temperature to block non-specific antigens, and incubated overnight with the primary antibodies at 4 °C: β-amyloid antibody (sc-28365; 1:50, Santa Cruz Biotechnology, USA), followed by fluorescence conjugated secondary antibody goat anti-mouse IgG (Anti-mouse IgG, 1:1000, #4408; CST, USA). The immunofluorescence was captured using an Olympus DP73 microscope. Quantitative image analysis was performed for Aβ immunofluorescence by taking micrographs of three sections per hippocampus and evaluated with Image-Pro Plus 6.0 software (Media Cybernetics, Rockville, Maryland, USA). The area fraction was the percentage of the area containing Aβ plaques in the hippocampal region in the total area of the hippocampal region.

### mRNA extraction and real-time PCR

Total RNA was taken from hippocampal tissue using a UNIQ-10 Column Trizol Total RNA Isolation kit (Sangon Biotech Co., Ltd, Shanghai, China) according to the manufacturer’s instructions. RNA was quantified using a NanoDrop One spectrophotometer (Thermo Scientific). RNA served as a template and the mRNA was converted to complementary DNA sequence by reversed transcription using a PrimeScript™ RT Master Mix 1st strand cDNA Synthesis Kit (Takara Bio Inc., Japan) according to manufacturer’s protocol. The cDNAs were dissolved in DEPC water and amplified using iTap Universal SYBR^@^ Green Supermix (Bio-Rad Laboratories, Inc., USA) and the following PCR primers: (1) BACE1, forward: ACTGCAAGGAGACGGAGAAGT, reverse: CGGCCGTAGGTATTGCTGAG; (2) HSF1, forward: CATGAGCCTGCCTGACCTG, reverse: CACCAGCTGCTTTCCTGAGT; (3) HSP70, forward: GAGATCGACTCTCTGTTCGAGG, reverse: GCCCGTTGAAGAAGTCCTG; (4) Tau, forward: GTCCTCGCCTTCTGTCGATT, reverse: GCTGTGGGGGAGACTCTTTT; (5) β-actin, forward: CCCCTGAACCCTAAGGCCA, reverse: CGGACTCATCGTACTCCTGC. RT-PCR amplification consisted of 40 cycles of 30 s at 95 °C, 3 s at 95 °C, and 30 s at 60 °C.

### Western blotting

Hippocampal tissue (15–25 mg) was homogenized and lysed with RIPA lysis buffer (PMSF solution (PMSF and RIPA) was added at the ratio of 1:100 = before use; Solarbio Life Sciences, R0020, China). In brief, hippocampal tissue was loaded into homogenization tubes, the configured RIPA lysis solution and two or three magnetic beads were added, homogenized at 70 Hz for 30 s, repeated three times and the final mixture centrifuged at 4 °C for 10 min at 10,000×*g*. The supernatant was collected, and protein concentration was determined by the BCA protein assay kit (Solarbio Life Sciences, PC0020, China). Proteins were separated on 4–12% Sure- PAGE™ and electrophoretically transferred to PVDF membranes that were then blocked with 5% defatted milk in TBST (0.1% Tween 20 in TBS) for 2 h at room temperature and incubated overnight at 4 °C with the following primary antibodies: PI3 Kinase p110β (ab151549, 1:1000, Abcam, USA), PI3 Kinase p85α (ab191606, 1:1000, Abcam, USA), AKT1 [ (phospho S473), ab81283, 1:1000, Abcam, USA], pan-AKT (ab18785, 1:2000, Abcam, USA), HSP70 (ab181606, 1:1000, Abcam, USA), BACE1 (ab183612, 1:1000, Abcam, USA), STUB1/CHIP (ab134064, 1:10,000, Abcam, USA), PGP9.5 (ab108986, 1:10000, Abcam, USA), P-Tau[(Ser202), #39357, 1:1000, CST, USA], P-Tau[(Thr181), #12885, 1:1000, CST, USA], Aβ (sc-28365, 1:1000, Santa Cruz, USA), β-actin (bs-0061, 1:1000, Bioss, Beijing, China). Membranes were rinsed, incubated for 1 h at room temperature with horseradish peroxidase (HRP) conjugated-secondary antibodies, respectively: Goat Anti-Mouse IgG H&L/HRP (bs-40296G-HRP, 1:10000, Bioss, Beijing, China), Goat Anti-Rabbit IgG H&L/HRP (bs-40295G-HRP, 1:10000, Bioss, Beijing, China). Membranes were developed with a chemiluminescent HRP substrate kit (WBKLS0500, Millipore, USA) and the protein bands on the membranes were densitometrically scanned by a gel image processing system (Amersham Imager 680, CTL, USA). The density of the protein bands was quantified using Image J software Version 1.8 (National Institutes of Health, Bethesda, MD, USA).

### Statistical analysis

All statistical analyses were performed using GraphPad Prism (version 8.0, La Jolla, California, USA). Two-way analysis of variance (ANOVA) (with exercise treatment and genotype as factors) was used for evaluation of differences between groups. The escape latency and percentage of time spent in the platform quadrant were analyzed using a repeated-measures ANOVA in the place navigation test. Post hoc tests were conducted to find out more about statistically significant ANOVA results. Statistical significance was considered if *P* < 0.05. All values are reported as means ± SEM.

## Results

### Treadmill exercise training alleviates MWM-based cognitive impairment and improves learning and memory ability in APP/PS1 transgenic mice

Previous reports have shown that 6-month-old APP/PS1 transgenic mice exhibit impaired learning and memory abilities [24]. To assess the therapeutic effect of treadmill exercise against cognitive decline associated with AD pathology, 3-month-old APP/PS1 transgenic mice were subjected to 12 weeks of treadmill exercise using Morris water maze (MWM) to assess spatial learning and memory abilities and memory retention. The overall experimental flow is shown in Fig. 1A.

With respect to MWM place navigation, comparison between groups revealed that the latency of mice in each group decreased gradually with the passage of time through the 5 days of learning as is shown in Fig. 1B. A repeated measurement ANOVA yielded a main effect of time [*F* (2.901, 20.31) = 49.66, *P* < 0.0001], a main effect of group [*F*(2.123, 14.86) = 20.17, *P* < 0.0001], but no significant effect of group by time interaction [*F*(3.810, 26.67) = 2.667, *P* = 0.0564]. Compared to normal wild-type mice, APP/PS1 control mice had a longer latency time (day 3 and day 5) with cognitive impairment (ADC vs. WTC, *P* < 0.01). However, in AD transgenic mice, exercise significantly reduced the latency from day 3 to day 5 (ADE vs. ADC, *P* < 0.05). Similarly, a repeated measurement ANOVA revealed significant main effects of time [*F*(2.840, 19.88) = 8.680, *P* < 0.001] and group [*F*(2.742, 19.19) = 21.65, *P* < 0.0001], but no significant effect of group by time interaction [*F*(4.551, 31.86) = 2.51, *P* = 0.6818] for the percentage of time spent in the platform quadrant (Fig. 1C). Post hoc analysis revealed that the percentage of time in the plateau quadrant was significantly reduced from day 4 to day 5 in the ADC group of mice compared to the WTC group (*P* < 0.05). In addition, in AD mice, exercise significantly increased the percentage of time in the platform quadrant on days 4 and 5 (ADE vs. ADC, *P* < 0.05). The results of this study suggested that exercise could improve learning memory in AD mice.

As with the performance during place navigation trials, there was also significant difference between groups during the space probe test on day 6. The number of platform crossings (Fig. 1D) during the probe test by two-way ANOVA revealed a main effect of exercise [*F*(1,28) = 4.431, *P* < 0.05], but no significant effects of genotype [*F*(1,28) = 3.172, *P* = 0.0858] and genotype × exercise interaction [*F*(1,28) = 1.285, *P* = 0.2666]. Post hoc analysis test revealed that the number of platform crossings was significantly decreased in the transgenic mice compared with wild-type control mice (ADC vs. WTC, *P* < 0.05). Interestingly, treadmill exercise significantly increased the number of platform crossings in ADC mice (ADE vs. ADC, *P* < 0.05). Two-way ANOVA suggested that there were significant main effects of genotype [*F*(1,28) = 12.28, *P* < 0.01; and *F*(1,28) = 23.54, *P* < 0.0001, respectively] and exercise [*F*(1,28) = 5.572, *P* < 0.05; and *F*(1,28) = 12.76, *P* < 0.01, respectively], but no significant effects of genotype × exercise interaction [*F*(1,28) = 0.05001, *P* = 0.8247; and *F*(1,28) = 0.004541, *P* = 0.9468, respectively] for the percentage of time spent (Fig. 1E) and the percentage of pathlength (Fig. 1F) in the platform quadrant. In addition, significant between-group differences were found in time spent in the target platform and distance swum. Mice in the ADE group spent longer time in the quadrant with the platform in a memory test conducted after the 5-day acquisition training compared to mice in the ADC group (ADE vs. ADC, *P* < 0.05). The ADE mice swam longer distances in the quadrant with a platform than the ADC mice (ADE vs. ADC, *P* < 0.01). Similarly, in wild-type mice, there was a similar trend in the time spent on the target platform and the distance swum (WTE vs. WTC, *P* < 0.05 and *P* < 0.01, respectively). The total swimming distances (Fig. 1G) by two-way ANOVA showed a main effect of genotype [*F*(1,28) = 9.599, *P* < 0.01], but no significant effects of exercise [*F*(1,28) = 0.1819, *P* = 0.6730] and genotype × exercise interaction [*F*(1,28) = 0.4298, *P* = 0.5175]. Significant difference was found in total swimming distances between these groups, with a significant reduction in the total swimming distance in the WTE group compared to the WTC group (WTE vs. WTC, *P* < 0.05). The decrease of the total swimming distance in the exercise group suggested that they could quickly find a platform to rest, and that exercise improved the cognitive ability of mice. The results of this study suggested that exercise could enhance memory retention in AD mice. Together, the current findings indicated that APP/PS1 transgenic mice had cognitive deficits in the early stages of neuropathological development and that their spatial learning and memory abilities were significantly impaired, which could be reversed and prevented via a forced physical exercise in this AD mouse model.

### Treadmill exercise training improves pathological characteristics in APP/PS1 transgenic mice

**Fig. 2:**
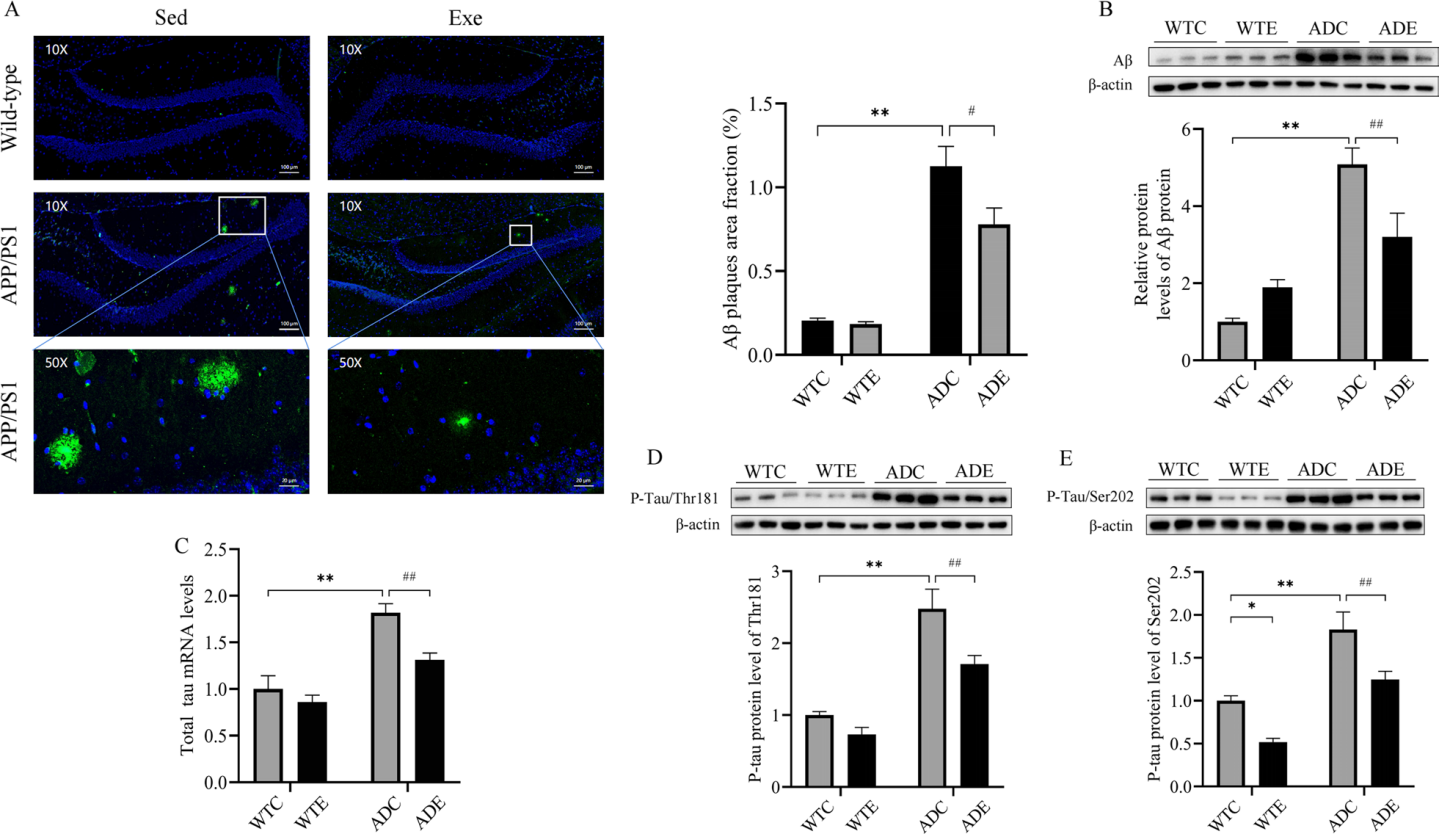
Effect of treadmill exercise on pathological characteristics of wild-type and APP/PS1 transgenic mice. **A** Immunofluorescence images showing Aβ plaques in the hippocampus of mice and statistical analysis of its data (*n* = 3 for each group). **B** Western blotting of Aβ and statistical analysis of its data (*n* = 6 for each group). **C** Statistical data showing the effect of exercise on hippocampal Total tau mRNA in each group of mice. **D** Western blotting of hippocampal p-Tau/Thr181 and statistical analysis of its data (*n* = 6 for each group). **E** Western blotting of hippocampal p-Tau/Ser202 and statistical analysis of its data (*n* = 6 for each group). Values are presented as mean ± SEM. Statistically different from WTC, *P < 0.05, **P < 0.01. Statistically different from ADC, ^#^*P* < 0.05; ^##^*P* < 0.01

To investigate the molecular mechanisms underlying the decline of learning and memory ability in APP/PS1 transgenic mice and the reversal effect of treadmill exercise, we quantified the area of Aβ plaques in hippocampal slices and the amount of Aβ peptide and P-Tau in the hippocampus, as is shown in the iconic image and the quantified results in Fig. 2A. Analysis of the area of Aβ plaques with two-way ANOVA showed main effects of genotype [*F*(1,8) = 95.87, *P* < 0.0001] and exercise [*F*(1,8) = 5.580, *P* < 0.05], but no significant effects of genotype × exercise interaction [*F*(1,8) = 4.409, *P* = 0.0690]. Post hoc analysis test revealed that ADC mice had a significantly higher Aβ plaque area than WTC mice (*P* < 0.01). The area of Aβ plaques was significantly reduced in ADE mice compared with ADC mice (*P* < 0.05). These results suggested that treadmill exercise reduced the level of Aβ deposition in APP/PS1 mice.

To further explore the effects of treadmill exercise on hippocampal Aβ, we measured the levels of Aβ peptides in the hippocampus (Fig. 2B). Analysis of Aβ peptide with two-way ANOVA revealed significant main effects of exercise [*F*(1,8) = 48.11, *P* = 0.0001] and genotype × exercise interaction [*F*(1,8) = 12.73, *P* < 0.01], but no significant effects of genotype [*F*(1,8) = 1.623, *P* = 0.2385]. With respect to the pathologic hallmarks of the disease, the level of hippocampal Aβ peptide was significantly higher in ADC mice than in WTC mice (*P* < 0.01). On the other hand, ADE mice had significantly lower levels of hippocampal Aβ peptide than WTC mice (*P* < 0.01). The above findings showed that exercise significantly reduced the abnormal accumulation of Aβ in the hippocampus.

In AD brains [25], Tau protein is phosphorylated mainly at Ser202, Ser396, Ser404 and Thr181 and Thr231 sites. In this study, the p-Tau levels of Tau protein at Ser202 and Thr181 sites were selected to detect the phosphorylation status of Tau protein. In response to the pathological features of AD, changes in hyperphosphorylated Tau protein levels reflect the extent to which exercise improves AD. As shown in Fig. 2C, analysis of Tau mRNA levels with two-way ANOVA revealed significant main effects of genotype [*F*(1,8) = 10.30, *P* < 0.05] and exercise [*F*(1,8) = 39.92, *P* < 0.001], but no significant effects of genotype × exercise interaction [*F*(1,8) = 3.281, *P* = 0.1077]. Mice in the ADC group had significantly higher total Tau mRNA levels than normal wild-type mice (ADC vs. WTC, *P* < 0.01), and exercise significantly reduced total Tau mRNA levels compared to mice in the ADC group (ADE vs. ADC, *P* < 0.01). Similarly, we made a similar observation at the protein expression level. Two-way ANOVA indicated that there were significant main effects of genotype [*F*(1,8) = 10.80, *P* < 0.05; and F(1,8) = 20.12, *P* < 0.01, respectively] and exercise [F(1,8) = 60.47, *P* < 0.0001; and F(1,8) = 43.03, *P* < 0.001, respectively], but no significant effects of genotype × exercise interaction [*F*(1,8) = 2.517, *P* = 0.1513; and *F*(1,8) = 0.1790, *P* = 0.6833, respectively] for the p-Tau proteins at Thr181 (Fig. 2D) and Ser202 sites (Fig. 2E). Post hoc analysis test revealed that the levels of hyperphosphorylated Tau protein were significantly increased in the ADC group mice compared to the WTC group mice (ADC vs. WTC, *P* < 0.01), mainly due to the abnormal accumulation of p-Tau proteins at Thr181 and Ser202 sites in the hippocampal region under AD pathology. In addition, treadmill exercise significantly suppressed the expression of p-Tau proteins at Thr181 and Ser202 sites compared to AD sedentary mice (ADE vs. ADC, *P* < 0.05). In conclusion, these findings suggested that mandatory exercise at an early stage of AD might reduce Aβ peptide production, alleviate Aβ plaque burden and reverse the effects caused by excess Aβ peptide in APP/PS1 transgenic mice. In addition, mandatory exercise training in the early stages of AD seemed to reduce the damage caused by neurofibrillary tangles.

### Treadmill exercise training activates the PI3K/Akt signaling pathway in the hippocampus of APP/PS1 transgenic mice

**Fig. 3:**
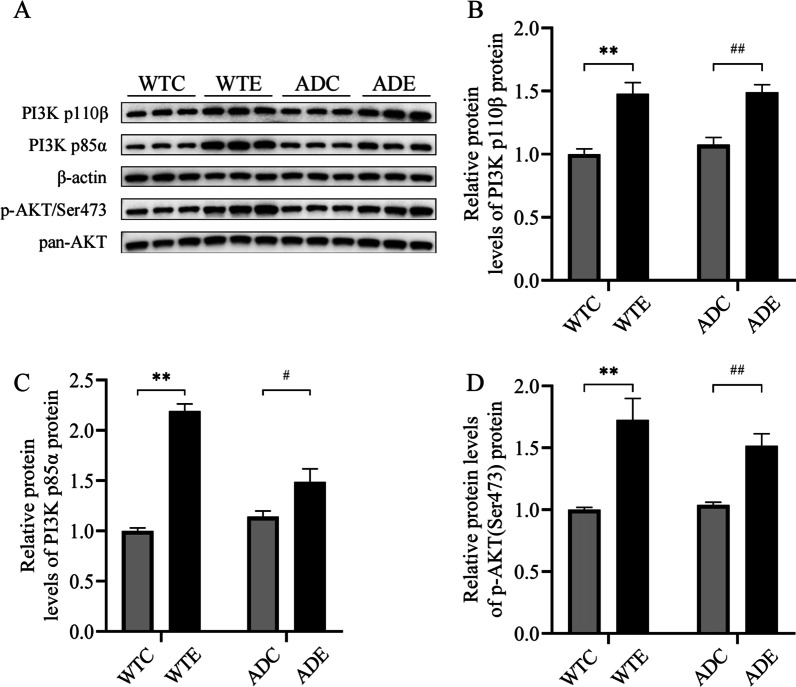
Effect of treadmill exercise on the PI3K/Akt signaling pathway in the hippocampus of wild-type and APP/PS1 transgenic mice (*n* = 6 for each group). **A** Western blotting of hippocampal PI3K p110β, PI3K p85α and p-Akt/Ser473. **B**–**D** Statistical data showing the effect of exercise on hippocampal PI3K p110β, PI3K p85α and p-Akt/Ser473 in each group of mice. Values are presented as mean ± SEM. Statistically different from WTC, **P < 0.01. Statistically different from ADC, ^#^*P* < 0.05; ^##^*P* < 0.01

PI3K is a member of the intracellular phosphatidylinositol kinase family that is composed of the catalytic subunit P110β and the regulatory subunit P85α, which activates Akt and regulates biological processes. Akt is a Ser/Thr protein kinase that translocates from the cytoplasm to the cell membrane via PI3K signaling, leading to phosphorylation of the threonine-308 site and the serine-473 site [26]. To assess the effect of treadmill exercise on the PI3K/Akt signaling pathway in the mouse hippocampus, we proceeded to find out whether there were alterations in the expression level of PI3K/Akt. Two-way ANOVA yielded that there were main effects of genotype [*F*(1,8) = 48.65, *P* = 0.0001; and *F*(1,8) = 36.23, *P* < 0.001, respectively], but no significant effects of exercise [*F*(1,8) = 0.4703, *P* = 0.5122; and *F*(1,8) = 0.7162, *P* = 0.4220, respectively] and genotype × exercise interaction [*F*(1,8) = 0.2533, *P* = 0.6283; and *F*(1,8) = 1.526, *P* = 0.2518, respectively] for the PI3K p110β (Fig. 3B) and p-Akt (Fig. 3D). Analysis of the level of PI3K p85α (Fig. 3C) with two-way ANOVA revealed significant main effects of genotype [*F*(1,8) = 92.44, *P* < 0.0001] and exercise [*F*(1,8) = 12.26, *P* < 0.01], as well as a significant genotype × exercise interaction [*F*(1,8) = 27.98, *P* < 0.001]. As shown in Fig. 3B and C, exercise significantly increased the level of PI3K p110β and PI3K p85α compared with sedentary mice (ADC vs. ADE, *P* < 0.01 and *P* < 0.05, respectively) in APP/PS1 transgenic mice. Similarly, in APP/PS1 transgenic mice, the phosphorylation levels of Akt followed the same trend as PI3K. Exercise significantly increased p-Akt expression in ADE mice compared to ADC mice (ADC vs. ADE, *P* < 0.01). Furthermore, treadmill exercise significantly increased the levels of PI3K p110β, PI3K p85α and p-Akt in WTE mice compared to WTC mice (WTE vs. WTC, *P* < 0.01 for all three proteins). However, it should be noted that levels of PI3K p110β, PI3K p85α and p-Akt were not decreased in pathological states in APP/PS1 transgenic mice relative to WTC mice and no significant differences were observed (ADC vs. WTC, *P* > 0.05 for all three proteins). These results suggested that a forced physical exercise at an early stage of AD might lead to activation of the PI3K/Akt signaling pathway and initially exert an anti-AD effect.

### Treadmill exercise training increases HSP70 and CHIP levels in the hippocampus of APP/PS1 transgenic mice

**Fig. 4:**
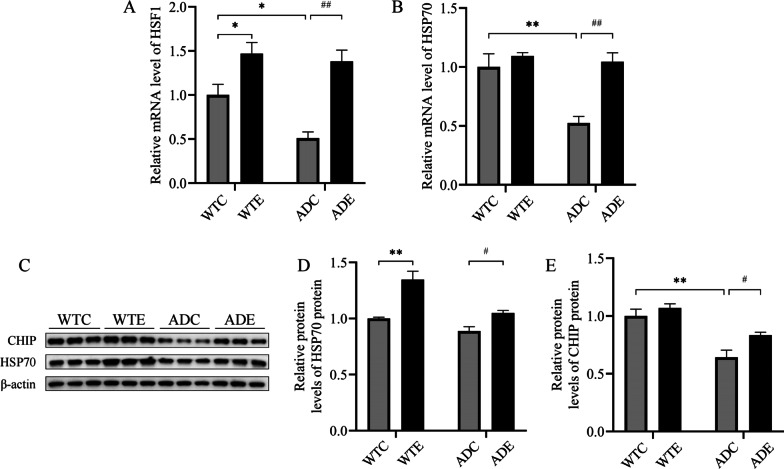
Effect of treadmill exercise on E3 ubiquitin ligase in the hippocampus of wild-type and APP/PS1 transgenic mice (*n* = 6 for each group). **A, B** Statistical data showing the effect of exercise on hippocampal HSFI and HSP70 mRNA in each group of mice. **C** Western blotting of hippocampal CHIP and HSP70. **D**, **E** Statistical data showing the effect of exercise on hippocampal HSP70 and CHIP in each group of mice. Values are presented as mean ± SEM. Statistically different from WTC, *P < 0.05, **P < 0.01. Statistically different from ADC, ^#^*P* < 0.05; ^##^*P* < 0.01

To further explore the mechanism by which exercise promotes UPS to improve the pathological characteristics of APP/PS1 transgenic mice, we examined the changes in CHIP and HSP70 in the hippocampus of each group of mice after compulsory exercise. Studies have already shown that levels of HSF1 and HSP70 are suppressed in the hippocampus of AD mice in pathological states, and that impaired UPS leads to reduced levels of CHIP, which is unable to clear accumulated abnormal proteins and exacerbates the condition. Two-way ANOVA yielded that there were main effects of genotype [*F*(1,8) = 35.24, *P* < 0.001; and *F*(1,8) = 16.88, *P* < 0.01, respectively] and exercise [*F*(1,8) = 6.497, *P* < 0.05; and *F*(1,8) = 12.37, *P* < 0.01, respectively], as well as a significant genotype × exercise interaction [*F*(1,8) = 6.524, *P* < 0.05; and *F*(1,8) = 8.102, *P* < 0.05, respectively] for the mRNA levels of HSF1 (Fig. 4A) and HSP70 (Fig. 4B). Our results are consistent with studies showing reduced mRNA levels of HSF1 and HSP70 in the hippocampus of AD-pathologized mice compared to wild-type mice (ADC vs. WTC, *P* < 0.05 and *P* < 0.01, respectively). In APP/PS1 transgenic mice, exercise significantly increased mRNA expression levels of HSF1 and HSP70 in the hippocampus compared to sedentary mice (ADE vs. ADC, *P* < 0.01). Two-way ANOVA yielded that there were main effects of genotype [*F*(1,8) = 32.30, *P* < 0.001; and *F*(1,8) = 7.549, *P* < 0.05, respectively] and exercise [*F*(1,8) = 20.96, *P* < 0.01; and *F*(1,8) = 38.53, *P* < 0.001, respectively], but no significant effects of genotype × exercise interaction [*F*(1,8) = 4.265, *P* = 0.0728; and *F*(1,8) = 1.613, *P* = 0.2398, respectively] for the protein levels of HSF1 (Fig. 4D) and HSP70 (Fig. 4E). Similarly, exercise significantly increased the expression level of HSP70 protein in the hippocampus of ADE group mice compared to ADC mice (ADE vs. ADC, *P* < 0.05). Furthermore, in wild-type mice, exercise also significantly increased the expression of HSP70 in the WTE group mice compared to the WTC group mice (WTE vs. WTC, *P* < 0.01). CHIP levels were significantly reduced in the hippocampus of APP/PS1 transgenic mice compared to normal wild-type mice (ADC vs. WTC, *P* < 0.01). Interestingly, exercise was able to significantly increase CHIP protein expression levels in the hippocampus of ADE group mice compared to ADC group mice (ADE vs. ADC, *P* < 0.05).

### Treadmill exercise training reduces BACE1 levels and increases UCHL-1 levels in the hippocampus of APP/PS1 transgenic mice as correlated events

**Fig. 5:**
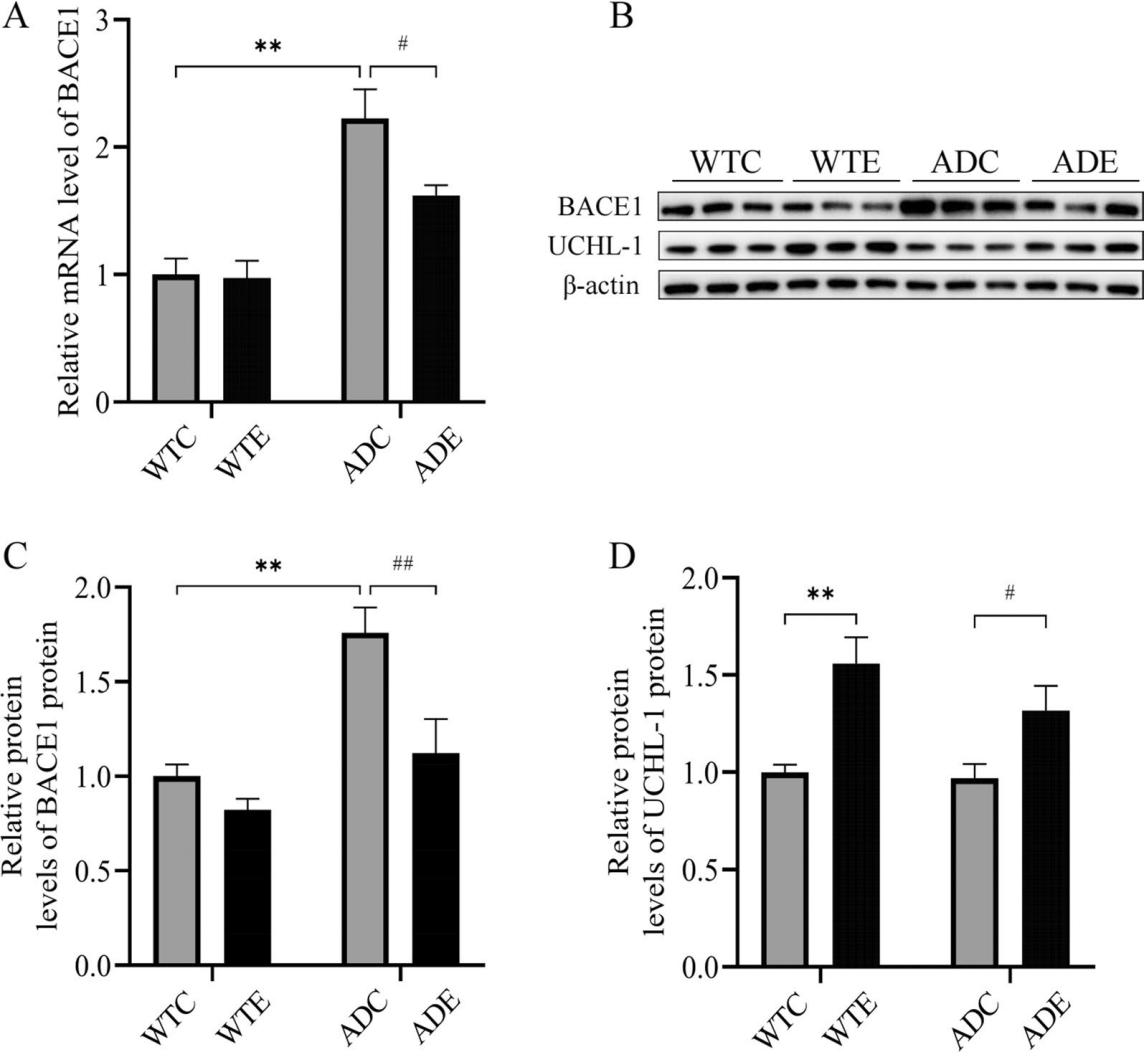
Effect of treadmill exercise on the BACE and UCHL-1 in the hippocampus of wild-type and APP/PS1 transgenic mice (*n* = 6 for each group). **A** Statistical data showing the effect of exercise on hippocampal BACE1 mRNA in each group of mice. **B** Western blotting of hippocampal BACE1 and UCHL-1. **C, D** Statistical data showing the effect of exercise on hippocampal BACE1 and UCHL-1 in each group of mice. Values are presented as mean ± SEM. Statistically different from WTC, **P < 0.01. Statistically different from ADC, ^#^*P* < 0.05; ^##^*P* < 0.01

Previous studies have established that changes in UCHL-1 levels are negatively correlated with BACE1 in AD brains. To investigate whether exercise-mediated changes in BACE1 affect UCHL-1 levels, we examined the expression of BACE1 and UCHL-1 following exercise. As shown in Fig. 5A, analysis of the BACE1 mRNA levels with two-way ANOVA revealed significant main effects of exercise [*F*(1,8) = 38.29, *P* < 0.001], but no significant effects of genotype [*F*(1,8) = 4.332, *P* = 0.0710] and genotype × exercise interaction [*F*(1,8) = 3.644, *P* = 0.0927]. Mice in the ADC group had very significantly higher BACE1 mRNA levels compared to normal wild-type mice (ADC vs. WTC, *P* < 0.01). Exercise significantly reduced BACE1 mRNA levels compared to mice in the ADC group (ADE vs. ADC, *P* < 0.05). Regarding protein expressions, two-way ANOVA yielded that there were main effects of genotype [*F*(1,8) = 11.52, *P* < 0.01; and *F*(1,8) = 19.94, *P* < 0.01, respectively] and exercise [*F*(1,8) = 19.46, *P* < 0.01; and *F*(1,8) = 11.79, *P* < 0.05, respectively], but no significant effects of genotype × exercise interaction [*F*(1,8) = 3.666, *P* = 0.0919; and *F*(1,8) = 1.086, *P* = 0.3278, respectively] for the protein levels of BACE1 (Fig. 5C) and UCHL-1 (Fig. 5D). Treadmill exercise significantly inhibited BACE1 protein expression in the hippocampus of AD transgenic exercise group mice relative to APP/PS1 transgenic quiet group mice (ADE vs. ADC, *P* < 0.01) (Fig. 5C). However, in wild-type mice, exercise did not significantly reduce BACE1 protein levels relative to the sedentary group of mice (WTE vs. WTC, *P* > 0.05). Interestingly, long-term exercise training significantly increased the protein expression of UCHL-1 in the hippocampus of AD transgenic exercise group mice compared to AD transgenic sedentary group mice (ADE vs. ADC, *P* < 0.05). Similarly, in wild-type mice, exercise was also able to upregulate UCHL-1 protein expression compared to wild-type sedentary mice (WTE vs. WTC, *P* < 0.01). These studies suggested that exercise reduced the secretion of BACE1, which in turn reduced the production of Aβ, regulated the expression of UCHL-1 and helped improve the cognitive and memory abilities of AD mice.

## Discussion

There is evidence that Aβ toxicity can exacerbate cognitive impairment and accelerate the course of AD [27–29]. Herein, our study found severely impaired spatial learning and memory performance (increased escape latencies, decreased percentage of time and distance spent in the platform quadrant and numbers of platform crossings) when measured with Morris water maze in 6-month-old APP/PS1 transgenic mice, paralleling an increased area of Aβ plaques in the hippocampus, which is consistent with previous findings [30, 31]. There is a large body of evidence that as a non-pharmacological means, exercise can effectively reduce Aβ deposition in the brain of AD mice [32, 33], improve learning and memory capacity [34, 35], delay cognitive decline [36] and inhibit the pathological process of AD [37]. Clinical studies have shown that physical exercise is effective in maintaining executive function in patients with mild AD [38], significantly relieving cognitive impairment in patients with mild AD [39, 40] and reducing the risk of developing AD. Interestingly, our findings found that 12 weeks of treadmill exercise reversed cognitive dysfunction in APP/PS1 transgenic mice (decreased escape latency, increased percentage of time and distance spent in the platform quadrant and numbers of platform crossings) and was accompanied by a reduction in the number of Aβ plaques in the hippocampus. Moreover, we and other studies [41] have also shown that treadmill exercise reduced soluble Aβ peptide levels in the hippocampus.

Impaired UPS function can lead to aggregation and hyperphosphorylation of Tau proteins, and hyperphosphorylated Tau proteins in turn inhibit UPS function [42, 43]. Our study found that phosphorylation levels of Tau Ser202 and Thr181 sites were significantly elevated in the hippocampus of APP/PS1 transgenic mice, while p-Tau levels were significantly reduced in the hippocampal region of AD transgenic mice after 12 weeks of mandatory exercise. This suggested that exercise reduced p-Tau levels and improved the pathological state of AD. The current findings conform with previous studies in which treadmill running suppressed tau phosphorylation levels at Ser404, Ser202, and Thr231 residues in the hippocampus of AD mice [21]. On the other hand, 3 months of treadmill exercise inhibited the expression of P-tau protein at Ser202, Ser404, Ser396 and Thr231 sites in the hippocampus of AD mice by upregulating the expression of phosphorylated PI3K and Akt [19]. Thus, our study also confirmed that exercise activated the PI3K/Akt signaling pathway, reduced p-Tau levels and improved cognitive impairment. The current findings support and extend previous research that physical exercise has a preventive and/or suppressive effects on cognitive decline in AD animal models.

We proceeded to investigate possible signaling pathways involved in the treadmill exercise-induced beneficial effects. Aβ is generated by sequential cleavages of β-amyloid precursor protein (APP) by BACE1 and γ-secretase. Studies have confirmed that as AD worsens, Aβ expression increases and Aβ oligomers inhibit the activation of the PI3K/AKT signaling pathway, leading to a gradual decrease in the phosphorylation expression levels of PI3K subunits and Akt, further causing an increase in BACE1 and PS1 expression, forming a vicious cycle [44]. Our results illustrated that inhibition of the PI3K/Akt signaling pathway in hippocampal tissue of APP/PS1 mice raised BACE1 levels, leading to an increase in the number of Aβ plaques. It was found that treadmill exercise activated the PI3K/Akt pathway in the hippocampus of AD model mice, upregulating p-PI3K and p-Akt levels [45]. After 12 weeks of treadmill training, PI3K p110β and p85α subunit protein levels were significantly upregulated in the hippocampal tissue of the brains of APP/PS1 exercise group mice, and phosphorylation levels at the Akt Ser473 site were also significantly increased, accompanied by a decrease in BACE1 levels. Previous studies have shown that treadmill exercise significantly reduced the expression levels of BACE1 and PS1 in the hippocampal tissue of APP/PS1 mice [46]. In addition, some studies [47] have reported that pharmacological activation of the PI3K/Akt signaling pathway in the cerebral cortex reduces the levels of BACE1 and γ-secretase, thereby reducing the formation of Aβ. The combination of data from different studies implies that treadmill exercise might effectively inhibit Aβ deposition by enhancing PI3K/Akt signaling pathway activity in the hippocampus of AD model mice and reducing the expression level of BACE1.

AD patients have UPS dysfunction. Accumulating evidence indicated that soluble UCHL-1 activity and protein levels are down-regulated and BACE1 levels are upregulated in the brains of postmortem AD patients and in APP/PS1 mouse models [3]. In our study, we also observed significantly elevated gene and protein levels of BACE1 in the hippocampus of APP/PS1 transgenic mice, accompanied by a significant suppression of UCHL-1 protein expression in the AD pathological state. Overexpression of UCHL-1 accelerates BACE1 degradation, impairs APP processing and reduces Aβ production [48]. Interestingly, protein levels of UCHL-1 were significantly increased and accompanied by a decrease in BACE1 levels in the hippocampus of APP/PS1 transgenic mice after exercise. Similarly, treadmill exercise also increased UCHL-1 expression levels in the hippocampus of normal wild-type mice. We speculated that this was possibly because exercise decreased BACE1 levels, leading to a corresponding compensatory increase in UCHL-1 levels. It is unclear whether there is a direct activation relationship between exercise and UCHL-1 or whether there are other factors that influence changes in UCHL-1 levels, and further studies are needed to solve these doubts. With respect to cognitive impairments, UCHL-1 was found to be required for neuronal synapse formation and maintenance of cognitive function. UCHL-1-deficient mice showed decreasing acetylcholine release from the synaptic terminal, reduced ubiquitin recycling and disruption of ubiquitin-dependent pathways, accompanied by hindered synaptic plasticity, nerve terminal retraction and axonal degeneration. Further studies [49] also revealed that overexpression of UCHL-1 repaired situational memory deficits and reestablished synaptic activity in APP/PS1 mice by reducing BACE1 levels. These data suggest that the lower expression of UCHL-1 may be partially responsible for cognitive impairment and AD pathophysiology. Based on our current data, it is at least partially illustrated that (exercise lowering BACE1 levels) may indirectly affect (upregulate) UCHL-1 levels and improve cognitive effect. Therefore, we hypothesized that exercise activated the PI3K/Akt signaling pathway in the hippocampus of APP/PS1 mice, decreased the expression level of BACE1 and correspondingly increased the level of UCHL-1, thereby improving cognitive impairment in the AD state.

Studies have shown that HSF1 and HSP70 are down-regulated in the AD brain [6, 8]. In addition, reduced HSP70 expression in AD brain was also accompanied by a significant decrease in Akt phosphorylation levels [17], suggesting inhibition of the PI3K/Akt signaling pathway in AD brain, increased Aβ misfolding and aggregation, and impaired Aβ clearance. Based on their strong pathological association with AD, we found that mRNA levels of HSF1 and HSP70 and protein content of HSP70 were significantly down-regulated in the hippocampus of APP/PS1 transgenic mice. Early studies have shown that the PI3K/Akt pathway regulates HSF1 gene expression, induces a heat shock response, induces increased HSP70 transcription, inhibits Aβ misfolding and aggregation, protects neuronal cells from stress-induced protein denaturation resulting in damage, and attenuates AD [50]. Another study revealed that exercise activated the PI3K/Akt signaling pathway in the brain of AD mice to upregulate HSP70 expression levels and decrease p-Tau content [21]. In line with this study, exercise was found to significantly increase gene expression of HSF1 and protein content of HSP70 in the hippocampus of APP/PS1 transgenic exercise group mice compared to the ADC group in our study. The above findings implied that exercise activated the PI3K/Akt signaling pathway in the hippocampal tissue of AD mice, upregulated HSF1 and HSP70 gene expression and induced an increase in HSP70 expression, which inhibited the formation of Aβ plaques and reduced hyperphosphorylated Tau protein levels.

To further investigate the specific mechanism of Tau protein dephosphorylation, we focused on another key E3 ligase (CHIP) in the UPS. Previous studies have demonstrated that CHIP deficiency exacerbates the phosphorylation of Tau proteins in the brains of AD mice [7]. Our study also demonstrated a significant decrease in CHIP levels in the hippocampus of APP/PS1 transgenic mice, accompanied by an increase in p-Tau levels. UPS-dependent P-Tau clearance is mediated by the overexpression of HSP70, and increasing the levels or effects of molecular chaperones in the UPS can effectively clear pathogenic proteins [51]. HSP70 acts as a cofactor for CHIP and overexpression of the molecular chaperone HSP70 facilitates clearance of tau by UPS. It has been shown that sulforaphane induces clearance of Aβ and Tau proteins and rescues memory deficits by increasing the expression levels of HSP70 and CHIP in the brains of AD transgenic mice [52]. CHIP binds to HSP70 to form the CHIP–HSP70 complex, which recognizes pathologically phosphorylated Tau and ubiquitinates it, and then passes the ubiquitinated Tau (primarily in its phosphorylated form) to the proteasome for degradation, reducing cell death caused by pathologically phosphorylated Tau protein [53]. Interestingly, we found that exercise significantly increased the protein content of CHIP in the hippocampus of APP/PS1 mice, and we speculated that this was because exercise upregulated HSP70 expression through activation of the PI3K/Akt pathway in the hippocampus of AD mice, which compensated by increasing the protein level of CHIP, which in turn formed a Tau/HSP/CHIP aggregate that was proteasomally degraded, reducing soluble phosphorylated Tau deposition and NFT formation. This also implies that the phosphorylation state of tau is regulated by the Hsp70-based chaperone machinery and undergoes Hsp70/CHIP-dependent ubiquitination and proteasomal degradation.

## Conclusion

To conclude, this study demonstrates that 12 weeks of compulsory physical exercise in the early states of AD may serve as a non-pharmacological means to delay and/suppress AD-like cognitive impairments based on MWM, as well as AD progression, by alleviating Aβ plaque burden and Tau hyperphosphorylation levels, promoting UPS function, and reducing Aβ and p-Tau production. The mediation of these effects may involve PI3K/Akt/HSP70 signaling pathway molecules, which also highlights the clinical importance of early interventions in AD patients. Yet, the effect of forced physical exercise on the ubiquitin–proteasome system is not fully understood and a more robust phenotype of AD pathology needs to be established to further explore the exact mechanisms.

## Supplementary Information

**Additional file 1:** Treadmill Exercise Reduces Body Weight in APP/PS1 Transgenic Mice.

## Data Availability

Data and materials are available on request.

## Abbreviations

Aβ: Amyloid peptides-β
AD: Alzheimer’s disease
ADC: Transgenic sedentary
ADE: Transgenic exercise
Akt: Protein kinase B
APP: Amyloid precursor protein
BACE1: β-Secretase 1
CHIP: Carboxyl terminus of HSP70 interacting protein
HSF1: Heat shock factor 1
HSP70: Heat shock protein 70
NFTs: Neurofibrillary tangles
PI3K: Phosphatidylinositol 3-kinase
P-Tau: Hyperphosphorylated Tau protein
SP: Senile plaque
UCHL-1: Ubiquitin carboxyl-terminal esterase-1
UPS: Ubiquitin–proteasome system
WTE: Wild-type exercise
WTC: Wild-type sedentary
