# Supplementary material for: Treadmill exercise promotes E3 ubiquitin ligase to remove amyloid β and P-tau and improve cognitive ability in APP/PS1 transgenic mice
Source: J Neuroinflammation. 2022 Oct 4;19:243. doi: 10.1186/s12974-022-02607-7 (PMC9531430; doi:10.1186/s12974-022-02607-7)
Supplement: Supplementary file 1 — Additional file 1: Treadmill Exercise Reduces Body Weight in APP/PS1 Transgenic Mice. [file 12974_2022_2607_MOESM1_ESM.doc]

**Treadmill Exercise Reduces Body Weight in APP/PS1 Transgenic Mice**

With respect to effects of genotype and treadmill exercise on body weight (Fig. 1), a repeated measurement ANOVA showed a main effect of time [*F*(1.291, 14.20) = 332.0, *P*<0.0001], a main effect of group [*F*(2.435, 26.79) = 4.605, *P*<0.05] and significant group by time interaction [*F*(4.711, 51.83) = 8.493, *P*<0.0001]. Post-hoc analysis revealed no significant difference in baseline weight (month 3) between these four groups. Similarly, there was no significant change in the body weight of mice in the ADC group relative to the WTC group (from the 3-month till the 6-month). However, the body weight of mice in the ADE group was significantly lower than that in the ADC group from the 5-month till the 6-month (ADE vs. ADC, *P*<0.05). These results indicate that three months of treadmill exercise reduces body weight in APP/PS1 transgenic mice.


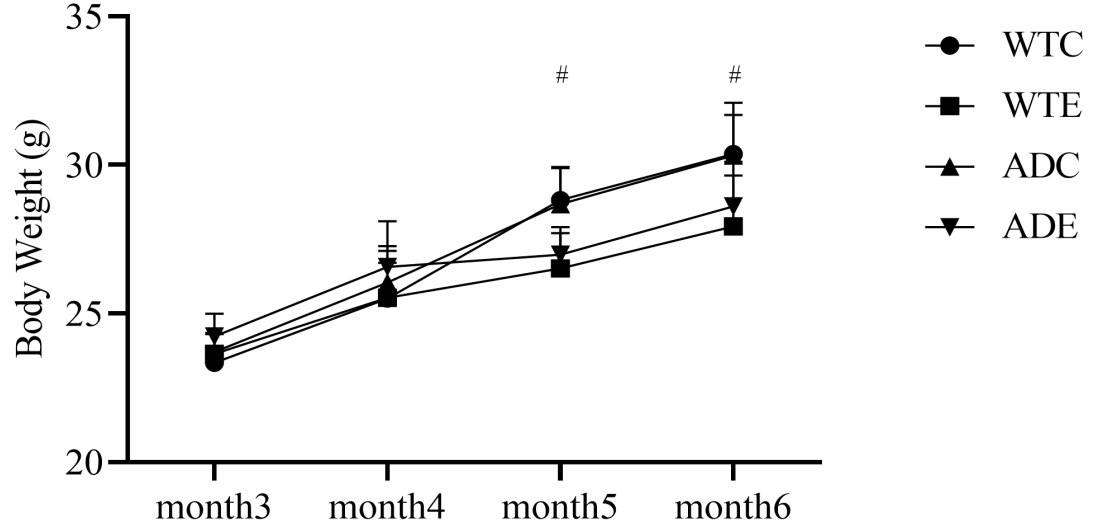


**Fig. 1** Effect of treadmill exercise on the body weight of wild-type and APP/PS1 transgenic mice. (n=12 for each group). Changes in body weight during 12-week treatment. Data are mean ± SEM. Statistically different between ADE and ADC, ^#^*P*<0.05.
